# The reasons and countermeasures of Bladder Rupture caused by Transurethral Clot Evacuation

**DOI:** 10.12669/pjms.37.3.3911

**Published:** 2021

**Authors:** Kai-long Liu, Xin Wang, Chang-bao Qu, Jin-Chun Qi

**Affiliations:** 1 Kai-long Liu, Department of Urology, The Second Hospital of Hebei Medical University, No. 215 Heping Xi Road, Shijiazhuang, Hebei, 050000, China; 2 Xin Wang, Department of Urology, The Second Hospital of Hebei Medical University, No. 215 Heping Xi Road, Shijiazhuang, Hebei, 050000, China; 3 Chang-bao Qu, Department of Urology, The Second Hospital of Hebei Medical University, No. 215 Heping Xi Road, Shijiazhuang, Hebei, 050000, China; 4 Jin-Chun Qi, Department of Urology, The Second Hospital of Hebei Medical University, No. 215 Heping Xi Road, Shijiazhuang, Hebei, 050000, China

**Keywords:** Bladder tamponade, Bladder rupture, Transurethral clot evacuation

## Abstract

**Objective:**

Bladder rupture caused by transurethral clot evacuation is rare in clinic, but an emergency operation is indeed needed in the patient with bladder rupture. We analyzed the reasons of bladder rupture caused by transurethral clot evacuation and provided the countermeasures to guide clinical surgeon to prevent the iatrogenic damage of bladder.

**Method:**

We retrospectively reviewed the records of 287 patients in our hospital, who had bladder tamponade resulting from clots of blood for various reasons and underwent transurethral clot evacuation from January 2007 to January 2019. Six male cases, aged from 28 to 76 years (mean 56.67±17.76) had bladder rupture. Four patients whose bladder ruptured intraperitoneally were changed to open surgery to repair bladder and clear the remanent blood clots. Two patients with extraperitoneal bladder rupture and a small bladder crevasse underwent a conservative therapy.

**Results:**

We observed that the incidence rate of bladder rupture was not associated with bladder tamponade and the age, but may be associated with gender, bladder paracentesis preoperative and urinary retention preoperative. All six cases were male.. They had different period of urinary retention before operation. No supra-pubis bladder paracentesis was made before operation. The bladder crevasses located in the triangle zone and posterior wall of bladder entirely, and the length of the bladder crevasses ranged from 3 to 7cm (mean 4.83cm). The bladder crevasses were all lengthways, and four cases were of’ bladders ruptured intraperitoneally while another two presented an extraperitoneal bladder rupture.

**Conclusions:**

The reasons of bladder rupture caused by transurethral clot evacuation may be related to gender, bladder paracentesis preoperative and urinary retention preoperative. We should decide to use expectant treatment or open surgery immediately according to the extent of the rupture when bladder rupture occurs.

## INTRODUCTION

Bladder rupture is the frequent acute abdomen of urology, and it’s usually caused by trauma, road accident, gunshot and iatrogenic operation.^1^-^6^ The bladder is the most common injury in genitourinary organ that from blunt pelvic trauma.^7^ Bladder trauma is seldom an isolated injury and it is frequently missed in poly-trauma cases because we pay more attention to life-threatening injuries like head injury, tension pneumothorax, hemothorax and hemoperitoneum.^2^,^8^,^9^ Bladder rupture caused by transurethral clot evacuation is rare in clinic, but an emergency operation is indeed needed in the patient with bladder rupture. In this study, we analyzed the reasons of patients with bladder rupture caused by transurethral clot evacuation, and provided the countermeasures to prevent the iatrogenic damage of bladder while doing the transurethral procedure using the endoscope.

## METHODS

The study was approved by the Institutional Ethics Committee on September 6^th^, 2020 from The Second Hospital of Hebei Medical University, and written informed consent was obtained from all participants.

We retrospectively reviewed the records of 287 patients, who had bladder tamponade resulting from clots of blood for various kinds reasons and underwent transurethral clot evacuation from January 2007 to January 2019. We noted six cases (2.1%) with bladder rupture. General information, such as age, gender, and cause of bladder tamponade, was collected for all patients. Bladder rupture in the six cases occurred when they underwent transurethral clot evacuation using cystoscopes sheath or resectoscope sheath and Ellik flusher. Supra-pubis bladder paracentesis hadn’t performed preoperatively. Of the 6 patients, one appeared bladder tamponade resulting from abundant of clots of blood when he engaged in out-of-bed activity after three days of percutaneous nephrolithotomy (PCNL), one appeared bladder tamponade because of tardive hemorrhage after seven days of PCNL, One presented witgh massive hemorrhage after 13 days of transurethral resection of prostate (TURP), two presented with massive hemorrhage within 48 hours of TURP, and the other one was spontaneous hemorrhage of prostate carcinoma. The bladder crevasses entirely located in the triangle zone and posterior wall of bladder, and the length of the bladder crevasses range from 3cm to 7cm (mean 4.83cm). There were all lengthways and four cases’ bladders ruptured intraperitoneally with a great quantity of seroperitoneum while another two cases presented an extraperitoneal bladder rupture and located in triangle zone only. Micturitional function and urine storage function of the 6 patients were not influenced finally.

### Surgical technique:

There were six patients who received transfusions based on their hemoglobin levels before transurethral clot removal. Two PCNL patients had undergone Transfemoral artery selective angio-embolization of renal artery before the transurethral clot evacuation. four patients whose bladder ruptured intraperitoneally were changed to open surgery to repair the bladder and clear the remanent blood clots. The peritoneal cavity was opened to clear the clots coming from the bladder crack and the seroperitoneum. The urinary catheter and bladder paracentesis catheter were detained simultaneously after operation. Two patients with extraperitoneal bladder rupture and a small bladder crevasse underwent a conservative therapy. Supra-pubis bladder paracentesis was performed, and then under the monitoring of cystoscopes, clot retention was successfully evacuated using the suction through the tunnel of bladder paracentesis. Ultimately, the urinary catheter and bladder paracentesis catheter were detained simultaneously. After operation, all the six patients were given continuous bladder irrigation, anti-inflammatory, hemostasis, fluid infusion and other supporting therapies. Bladder irrigation was suspended after one or two days when the rinse solution was not red. Three to five days after the operation, the catheter clipping was performed within 24 hours before the removal of the catheter to train the bladder function. However, the paracentesis catheter was reserved one month. Micturitional function and urine storage function of the six patients were not influenced finally.

## RESULTS

Bladder rupture occurred in 6 of 287 patients, and they were all male with their age from 28 to 76 years (mean 56.67±17.76). The analysis of the reasons of the clinical characteristics of bladder tamponade (Table-I), the correlation between the incidence rate of bladder rupture and the clinical characteristics variables of patients was evaluated. The incidence of bladder rupture was not associated with the reason of bladder tamponade and the age, but may be associated with gender, bladder paracentesis preoperative and urinary retention preoperative.

**Table I:** Characteristics of bladder tamponade

Clinical characteristics	Case no. (n=287)	Bladder rupture no. (n=6)
***Age***		
≤60	112	2
>60	175	4
***Gender***		
Male	219	6
Female	68	0
***Reason of Bladder Tamponade***		
PCNL	42	2
TURP	216	3
Other	29	1
***Urinary retention preoperative (Yes or No)***		
Yes	79	6
No	208	0
***Bladder paracentesis preoperative (Yes or No)***		
Yes	62	0
No	225	6

The analysis of the characteristics of the patients with bladder rupture caused by transurethral clot evacuation (Table-II), we observed that all six cases were male and the age had no obvious feature and had different extent urinary retention before operation. No Supra-pubis bladder paracentesis was performed preoperatively. The bladder crevasses located in the triangle zone and posterior wall of bladder entirely, and the length of the bladder crevasses range from 3 to 7cm (mean4.83cm). Four cases’ bladders ruptured intraperitoneally while another two presented an extraperitoneal bladder rupture.

**Table II:** Characteristics of bladder rupture.

Serial number	Age (year)	Sex	Reasons of bleeding	Urinary retention preoperative (yes or no)	Bladder paracentesis preoperative(yes or no)	Location of the bladder rupture	Length of the rupture (cm)	Intraperitoneal or extraperitoneal
1	28	Male	7 days after PCNL	Yes	No	Triangle zone and posterior wall	5	Intraperitoneal
2	43	Male	3 days after PCNL	Yes	No	Triangle zone and posterior wall	7	Intraperitoneal
3	76	Male	13 days after TURP	Yes	No	Triangle zone	3	extraperitoneal
4	61	Male	Bleed of Pca	Yes	No	Triangle zone and posterior wall	5	Intraperitoneal
5	65	Male	2 days after TURP	Yes	No	Triangle zone and posterior wall	4	Intraperitoneal
6	67	Male	1 days after TURP	Yes	No	Triangle zone	5	Extraperitoneal

## DISCUSSION

Bladder tamponade is a frequent complication of urologic disease and it must be managed urgently.^3^,^10^ Bladder tamponade will happen all sorts of urologic surgery including open surgery and endoscopy surgery.^2^,^11^,^12^ Blood clots will accumulate in bladder at the last, no matter bleeding appears in upper urinary tract or lower urinary tract, and this will result in bladder tamponade and urinary retention in succession, while a surgery is often indeed needed.^2^,^10^,^11^ Studies have reported rectal tube, catheter irrigating syringe, 6-hole irrigation catheter which were all successfully used for clot retention.^13^,^14^ Recently, with the enormous development of intracavity technique and equipment, the majority can be managed by transurethral clot evacuation using cystoscopes sheath or resectoscope sheath and Ellik flusher, except a few who underwent long time bleeding resulting in hematoma which need open surgery. It is easy to accept by patients because of its low side effects and no incision. Yu HS et al.^14^ reported a so-called “suction and fishing method” for severe clot retention.

As a result, all the patients’ blood clots was successfully eliminated. Others reported applying stronger suction pressure through the cystoscope sheath in patients who had failed clot evacuation using the traditional Ellik evacuator, and all patients were rendered clot free.^13^ The operation may lead to bladder rupture is a new problem.^15^,^16^ The incidence of bladder rupture was 2.1% in 287 patients enrolled in this study. The incidence of bladder rupture was not associated with the reason of bladder tamponade and the age, but may be associated with gender, bladder paracentesis preoperative and urinary retention preoperative. We conclude the following viewpoints through our analysis and summary:

1. The bladder rupture is usually induced by intraoperative misoperation.
2. Generous blood clots influence the legibility of the visual fields of endoscopy, and the violent suction while the sheath of cystoscopes holds out against the bladder wall, bladder rupture may appear finally.
3. Bladder tamponade will result in urine retention, and then muscle fibers of the bladder will be obviously damaged when bladder volume stretches rapidly. It may induce bladder injure more easily under the damage of external force.
4. It will accumulate more and more gas while we are using Ellik to wash bladder, and thus increase the tension of the bladder wall.
5. Bladder rupture often appears in the triangle zone and posterior wall on the ground that the sheath of cystoscopes is much easier to damage there during the processes of suction.
6. During our study we observed that the patients were all male, bladder rupture may relate to the long urethra of male, and thus might easily lead to hypertonic bladder induced by the accumulation of gas and fluid. It was difficult to observe hypertonic bladder when the patients were females because of their short urethra, so the probability of bladder rupture reduced greatly.

In view of the above we suggest that to avoid bladder rupture:

1. Decrease movement of cystoscopes sheath or resectoscope sheath to avoid physical damage to bladder.
2. We must release the majority of the gas and fluid in the bladder through cystoscopes sheath or resectoscope sheath to make sure we have a hypisotonic bladder.
3. Slight and multiple bladder irrigation using Ellik are indeed needed.
4. Don’t scruple to perform the supra-pubis bladder paracentesis because the little damage will bring about enormous bench. We not only always use it to keep a hypisotonic bladder throughout the operation, but also to settle the bladder tamponade by using the wall suction through the tunnel of bladder paracentesis under the monitoring of cystoscopes.

We should decide to use expectant treatment or open surgery immediately according to the extent of the rupture when bladder rupture occurs. Patients who have extraperitoneal bladder rupture but the bladder crevasses are small (less than 3cm) will undergo a conservative therapy. The urinary catheter and bladder paracentesis catheter are detained simultaneously to let bladder recover itself. However in those patients with big and/or intraperitoneal crevasses, the selection of open surgery is advisable. We cannot only handle their bladder ruptures and clear the remanent blood clots, but also clear the clots crack and the seroperitoneum coming from bladder simultaneously since the peritoneal cavity was opened.

### Limitations of this study:

Although 287 subjects were included in this study, only six cases could be found to analyze the factors related to bladder rupture, so the research conclusions drawn from this study were limited in persuasion. We look forward to start multi-center clinical studies in the future to further expand the sample size.

## CONCLUSIONS

The reasons of bladder rupture caused by transurethral clot evacuation may be associated with gender, bladder paracentesis preoperative and urinary retention preoperative. We should decide to use expectant treatment or open surgery immediately according to the extent of the rupture when bladder rupture occurs.

### Authors’ Contributions:

**KL L, XW** and **JC Q** designed and performed the experiments, prepared the manuscript, and are responsible and accountable for the accuracy or integrity of the work.

**CB Q** conducted the experiments and revised the manuscript.

All authors have read and approved the final manuscript.
